# Repeated Sprint Ability in Young Basketball Players: Multi-direction vs. One-Change of Direction (Part 1)

**DOI:** 10.3389/fphys.2016.00133

**Published:** 2016-04-22

**Authors:** Johnny Padulo, Nicola L. Bragazzi, Pantelis T. Nikolaidis, Antonio Dello Iacono, Giuseppe Attene, Fabio Pizzolato, Juliano Dal Pupo, Alessandro M. Zagatto, Marcello Oggianu, Gian M. Migliaccio

**Affiliations:** ^1^University eCampusNovedrate, Italy; ^2^Faculty of Kinesiology, University of SplitSplit, Croatia; ^3^Department of Health Sciences, University of GenoaGenoa, Italy; ^4^Department of Physical and Cultural Education, Hellenic Army AcademyAthens, Greece; ^5^Department of Life Science, The Zinman College for Physical Education and Sport ScienceWingate, Natanya, Israel; ^6^CONI – Italian Olympic CommitteeCagliari, Sardinia, Italy; ^7^Department of Neurological and Movement Sciences, University of VeronaVerona, Italy; ^8^Biomechanics Laboratory, Federal University of Santa CatarinaFlorianópolis, Brazil; ^9^Laboratoty of Physiology and Sports Performance, Physical Education Department, Faculty of Sciences, UNESP-Universidade Estadual PaulistaBauru, Brazil; ^10^Sport Science LabLondon, UK

**Keywords:** athletic performance, physical fitness, physical endurance, shuttle running, team sport

## Abstract

The aim of the present study was to examine the reliability of a novel multi-direction repeated sprint ability (RSA) test [RSM; 10 × (6 × 5-m)] compared with a RSA with one change of direction [10 × (2 × 15-m)], and the relationship of the RSM and RSA with Yo-Yo intermittent recovery test level 1 (Yo-Yo IR1) and jump performances [squat jump (SJ) and counter-movement-jump (CMJ)]. Thirty-six (male, *n* = 14, female *n* = 22) young basketball players (age 16.0 ± 0.9 yrs) performed the RSM, RSA, Yo-Yo IR1, SJ, and CMJ, and were re-tested only for RSM and RSA after 1 week. The absolute error of reliability (standard error of the measurement) was lower than 0.212 and 0.617-s for the time variables of the RSA and RSM test, respectively. Performance in the RSA and RSM test significantly correlated with CMJ and SJ. The best time, worst time, and total time of the RSA and RSM test were negatively correlated with Yo-Yo IR1 distance. Based on these findings, consistent with previously published studies, it was concluded that the novel RSM test was valid and reliable.

## Introduction

Basketball is characterized by high intensity short-duration activities (e.g., sprinting, jumping, passing, and shooting; Padulo et al., 2015a), interspersed by low-to-moderate intensity motion patterns (e.g., standing, walking, and jogging). It, therefore, seems that the ability to continuously perform intermittent high-intensity actions throughout the game is crucial for basketball players (Ben Abdelkrim et al., 2007). Time motion analyses (Ben Abdelkrim et al., 2007) have consistently reported that players perform repeatedly short sprints during the match, thus suggesting that success in basketball participation appears to be mostly dependent on the players' anaerobic metabolism and abilities (Padulo et al., 2015b). Accordingly, in addition to physical (e.g., stature) and physiological characteristics (Attene et al., 2015b), basketball players should also possess increased repeated sprint ability (RSA). The recognition of the important role of RSA for performance in basketball has led to the wide use of RSA-based tests among basketball players as part of routine fitness testing (Meckel et al., 2009; Caprino et al., 2012; Balsalobre-Fernández et al., 2014), as well as for training session strategy (Attene et al., 2015b).

RSA tests might vary according to sprint distance, number of sprints, duration and mode of recovery among sprints, and number and mode of change of direction (Girard et al., 2011). The existing RSA protocols include 10 × (2 × 15-m) with 30-s passive recovery (Caprino et al., 2012; Stojanovic et al., 2012), 12 × 20-m starting every 20-s (Meckel et al., 2009), 7 × 35-m with a slalom and 25-s active recovery (Carvalho et al., 2011), 6 × 35-m with 10-s active recovery (Zagatto et al., 2009; Balsalobre-Fernández et al., 2014) and 3 × (5 + 6 + 10 + 9-m) with 20-s recovery (te Wierike et al., 2014). Considering the official dimensions of a playing court (28-m in length and 15-m in width; FIBA, 2014), protocols using sprints of 35-m might be less relevant for basketball than those performed with smaller distances. Moreover, taking into account the numerous changes of direction occurring during match, a protocol with a change of direction [e.g., 10 × (15 + 15-m) with 30-s passive recovery] might be more sport-specific than those with no change of direction.

RSA test with a change of direction has been widely used to monitor performance in basketball (Caprino et al., 2012; Stojanovic et al., 2012; Attene et al., 2014; Padulo et al., 2015b). This test can be used to evaluate the ability to repeatedly sprint forwards and backwards, thus reflecting specific game situations that commonly occur during a basketball match (Caprino et al., 2012), such as the transitional movements from defense to offense and vice versa. Although the usage of RSA-based test has enhanced the understanding of sport-specific RSA, this test has addressed so far change of direction only with regards to forwards-backwards and not in lateral directions. However, numerous short sprints might occur in successive different directions, e.g., during offense when a basketball player moves from outside the three-point line toward the low post and back to the three-point line, and then moves laterally outside the three-point line or during man-to-man defense when a defender follows offender's actions. Therefore, the specific nature of basketball might require the development of a more sport-specific RSA with multi change of direction, i.e., sprint forwards and backwards, to the left and backwards, and to the right and backwards. The development of a multi change of direction is expected to help fitness trainers and coaches for talent identification, players' selection and training evaluation purposes, among others.

Nevertheless, multi change of direction should be firstly tested for reliability and validity before they can be routinely used by coaches and trainers. With regards to reliability of RSA protocols, very limited data are available from previous studies. The validity of RSA protocols has been previously examined comparing RSA indices (e.g., best time or BT, total time or TT, and fatigue index or FI) with performance measures such as Yo-Yo intermittent recovery level 1 (Yo-Yo IR1) and jumping tests (e.g., squat jump, SJ, and counter-movement jump, CMJ).

Yo-Yo IR1, which was developed as a measure to assess performance for team sport players, has been shown to largely correlate with maximal oxygen uptake (VO_2*max*_), speed at VO_2*max*_ and %VO_2*max*_ at ventilator threshold in young basketball players (Boullosa et al., 2013; Castagna et al., 2013; Rebelo et al., 2014). SJ and CMJ, which reflect different degrees of storage and reutilization energy (Bobbert et al., 1996), have been considered as measures of lower limbs' strength (Comfort et al., 2014; Baldi et al., in press). Further, TT of RSA-based test has been found to largely correlate with CMJ (Stojanovic et al., 2012; Attene et al., 2015a).

Therefore, the main purposes of the present study were to:

- assess the reliability of both multi change of direction and RSA tests,
- examine the physiological effects (blood lactate concentration or BLa and rating of perceived exertion or RPE) of multi change of direction with regards to RSA, and
- assess the relationship between multi change of direction and RSA tests with Yo-Yo IR1 and jump performance (SJ, CMJ).

The research hypotheses were that:

- multi change of direction, compared to RSA, is reliable as well, even though to a lesser extent,
- induces higher physiological impact (BLa and RPE) due to its movement-related nature
- exhibits correlation with Yo-Yo IR1, and
- presents correlation with SJ and CMJ, such as RSA.

## Methods

### Participants

Thirty-six young (Male, *n* = 14 and Female, *n* = 22) basketball players (age: 16 ± 1 yrs, height: 1.70 ± 0.10-cm and body mass: 60.0 ± 7.7 kg, BMI: 20.8 ± 1.8 kg.m^−2^, training experience 7.6 ± 1.2 yrs with ~5 h training per week) were recruited from San Paolo Basket Cagliari. All participants competed in the Under 17 Italian National Basketball Championship during the years 2012–2013.

Inclusion criteria to participate in the study were:

- participation at least 90% of the training sessions,
- regularly participating at the previous competitive seasons,
- having a valid sport medical clearance, and
- being healthy, not suffering from pain or injuries and not taking any drugs.

To reduce any interference on the experiment, participants were prohibited from consuming any known stimulant (e.g., caffeine) or depressant (e.g., alcohol) substances for 24-h before protocol and were instructed not to eat for 2–3 h before testing. To eliminate any influence of circadian variation (Ammar et al., 2015) each participant completed all trials at the same day-time (14/16 p.m.), in ambient conditions of 22.2 ± 0.5°C of temperature and 68.3 ± 3.5% of relative humidity. All tests were performed on a regular indoor basketball court and the participants wore basketball shoes and adequate sportswear. Participants (or their parents/guardians) gave their written consent, after being thoroughly informed of the purpose, benefits, and potential risks of the study. All experimental procedures were approved by the local University Human Research Ethics Committee of the CONI (Italian Olympic Committee), Sardinia, Cagliari (Italy), according to the ethical principles laid out in the 2008 revision of the Declaration of Helsinki.

### Experimental set-up

All participants performed four randomized testing sessions: (a) multi change of direction, (b) RSA, (c) jump performance, and (d) Yo-Yo IR1. Multi change of direction and RSA tests were repeated 1 week after the last testing session, in order to assess the reliability of the measures. Even though we used randomization in order to have reliable results, the lack of balanced order could be a study limitation. Further, although some authors (Buchheit et al., 2010) found that jumping during the recovery did not have an adverse effect on the ability to repeat sprints, in order to avoid any other side effect, tests were performed every 2 days paying a lot of attention in not accumulating fatigue. Before each test, participants completed a 10-min warm-up at low-intensity running (~8 km·h^−1^) and 5-min of standardized dynamic stretching (Chaouachi et al., 2015). For both tests (multi change of direction and RSA), 5 s before starting each sprint, participants assumed the ready position and waited for the starting signal (Padulo et al., 2012). The time for each single shuttle sprint was recorded using two sets of photocell gates (Brower Timing System, Salt Lake City, UT, USA; accuracy of 0.01-s) placed at a height of 50-cm on the starting and distinct finishing line as standard calibration (Padulo et al., 2014).

Participants were first familiarized with the test: they performed three sub-maximal 30-m shuttle sprints with 1-min of recovery in-between. After 5-min of recovery, a preliminary maximal single shuttle sprint was assessed as the criterion score for the subsequent 10 × 30-m shuttle sprint tests. Then, after 5-min of rest, the multi change of direction test or the RSA test started. When the performance in the first sprint during both tests was worse than the criterion score (i.e., an increase in time >2.5%), the test was immediately stopped and the participants were required to repeat the test with maximal effort after a 5-min rest.

### Repeated sprint ability test

The RSA test (Padulo et al., 2015b) consisted of ten 30-m shuttle-sprints (15 + 15-m) with a change of direction of 180°, interspersed by 30-s of passive recovery (walking back to the starting line and waiting for the next sprint), and with exercise to-rest-ratio of 1:5 (Ruscello et al., 2013). Participants started from the starting line, sprinted in-line for 15-m, touched a line on the floor with a foot and then came back with a 180° change of direction to the starting line as fast as possible. To balance the legs' physical effort during the change of direction, participants were asked to alternate the legs' use during each sprint (Pau et al., 2014).

### Repeated sprint multi-direction test

**Figure 1:**
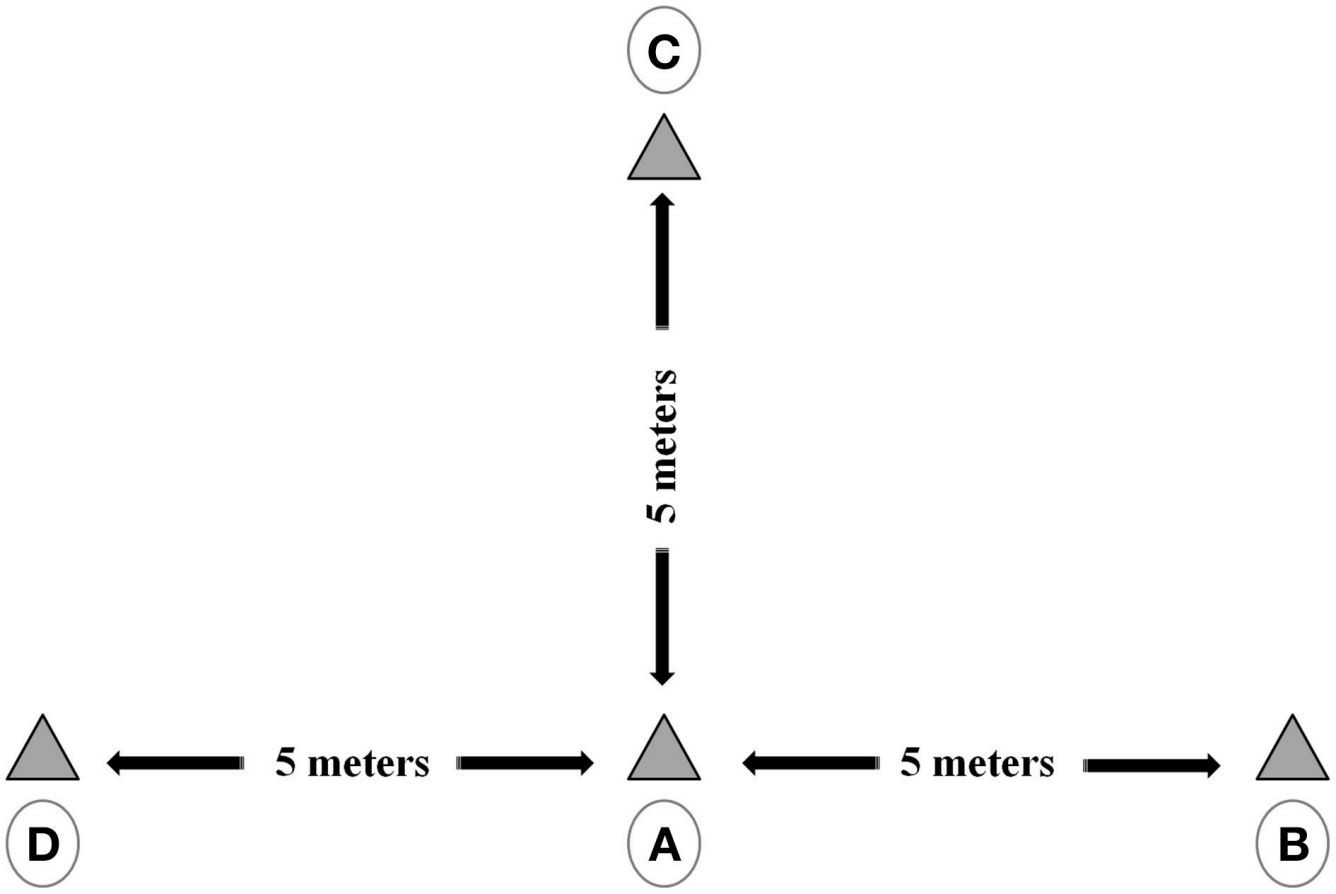
**Graphical representation of the multi change of direction (RSM) test**. Each shuttle started from the point A and the participant decided independently whether sprint toward the point B or the point D. After having touched the target line with one foot and they returned to the starting point A and subsequently, they had to necessarily sprint to the point C and back to the point A and finally sprint to the remaining point (D or B).

The repeated sprint multi-direction test (RSM) consisted of 10 repetition of 30-m shuttle sprints divided into six sprints of 5 m with five changes of direction (Figure 1), spaced out by 30-s of passive recovery (walking back to the starting line and waiting for the next sprint). Participant took place at the starting point A and decided independently whether to sprint toward the point B or the point D. For every shuttle, after 5 m, he/she touched the target line with one foot and had to return to the starting point A. Subsequently, he/she had to necessarily sprint to the point C and back to the point A and finally sprint to the remaining point (point D or B). The participants ran forward and, in order to balance the legs' effort during the change of direction, they were asked to alternate the legs' used every sprint (Pau et al., 2014).

### Jump performance tests

SJ and CMJ were explained and demonstrated to each participant and familiarization trials were completed until the correct technique was controlled. These tests were performed according to the protocol described by Bosco and Rusko (1983). Before each test session, participants performed a standardized warm-up routine that consisted of 5 min of jogging at a comfortable pace and 2 min of dynamic stretching of the lower extremity muscles (Haddad et al., 2014). All players performed 3 repetitions of both SJ and CMJ tests, with a passive rest of 1-min in-between, and 3-min between the two tests. Accelerometer Myotest™ (Myotest SA, Sion, Switzerland) was used (Choukou et al., 2014), to evaluate the height (cm) of vertical jump. For SJ, participants started from the upright standing position with their hands on their hips; they were, then, instructed to flex their knees and hold a predetermined knee position (~90°), for 3-s. At that point, participants were instructed to jump as high as possible, without performing any counter-movement (Padulo et al., 2013; Gheller et al., 2015). For CMJ, participants started from the upright standing position with their hands on their hips; they were, then, instructed to flex their knees (~90°) as quickly as possible and, then, jump as high as possible, with no delay in the bottom position. If a jump was incorrectly performed, participants were asked to repeat the trial.

### Yo-Yo intermittent recovery test

During Yo-Yo IR1, all players were already familiar with the testing procedures as it was part of their usual fitness assessment program (Padulo et al., 2015b). The Yo-Yo IR1 consisted of 20-m shuttle runs performed at increasing velocities with 10-s of active recovery between runs, until exhaustion. The test was considered to be over when the participant failed twice to reach the finishing line in time (objective evaluation) or when felt unable to complete another shuttle at the dictated speed (subjective evaluation). The total distance covered during the test (including the last incomplete shuttle) was considered as the test score.

### Rating of perceived exertion assessment and blood lactate sampling

Participants indicated their RPE using the category rating-10 (CR-10) scale, as modified by Foster et al. (2001), immediately at the end of each tests (SJ, CMJ, multi change of direction, RSA, Yo-Yo IR1). While blood lactate (BLa) concentration (mmol·L^−1^) was determined at third minute after the end of the multi change of direction, RSA and Yo-Yo IR1 tests were assessed as commonly reported in the extant scientific literature (Gharbi et al., 2008). Briefly, a micro-sample of arterialized blood from the ear lobe was taken and immediately analyzed with a lactate analyzer (Arkray Lactate Pro LT-1710—Kyoto, Japan).

### Statistical analysis

Mean ± SD of BT, WT, TT (sum of 10 sprints), FI, BLa, and RPE were computed for both the multi change of direction and RSA tests. In particular, FI was calculated following the Fitzsimons' formula (Fitzsimons et al., 1993):

$$100\;\cdot \;\frac{TT}{BT\cdot 10}-100$$

*A priori* power analysis was conducted with the program G^*^Power (Faul et al., 2007). The repeated measure ANOVA within between interaction with an effect size of at least 0.25, α = 0.05 and 1-β = 0.95 gave a statistical power of 95.17% and sample size of minimum 36 subjects. A statistical power of 95.17% and a sample size of 32 were detected with the minimum standardized regression coefficients equal to 0.75, α = 0.05 and 1-β = 0.95 with two-tailed distribution.

The normality of the distribution of the population was tested with the Shapiro–Wilk test and, before carrying out the ANOVA analysis, the homogeneity of variances was verified with the Bartlett's test.

Reliability (Hopkins, 2000) of each test was assessed by the two-way mixed single measures Intra-class Correlations Coefficient (ICC_3, 1_) (Bartko, 1966). The (ICC_3, 1_) assesses the consistency or reproducibility of quantitative measurements made by different observers measuring the same quantity where the “3” refers to the type of ICC in which the subjects is a random effect and the trials is a fixed effect, while the “1” refers to the reliability of single repeated measurements (not the mean of several measurements). This ICC is the correlation expected between the pairs of measurements in any of the two trials. An ICC of *r* = 0.80 represents good agreement, and a value *r* > 0.90 is considered to indicate excellent agreement (Donner and Eliasziw, 1987). The standard error of measurement (SEM) provides an indication of the dispersion of the measurement errors when you are trying to estimate the true scores from observed test scores. Finally, the reliability was tested with the Band-Altman plot (Bland and Altman, 2012). Moreover, we computed the *z*-value with this formula:

$$\text{z}=\frac{\sqrt{{}^{\text{Variability of the differences}}\text{​​}╱\text{​}{\text{​}}_{\text{number of participants}}\;}}{\text{mean of the differences}}$$

If the *z*-value is inside ±1.96 it means that the two measures are statistically equivalent, otherwise they are significantly different. A Pearson product-moment correlation coefficient was computed to assess the relationship between the RSA and multi change of direction, while, a Spearman's rank correlation was used for RPE. Furthermore, a three-way Analysis of Variance (ANOVA) was conducted, the within factors were the two types of tests (multi change of direction and RSA) and test/re-test condition, while the between factor was the gender (male and female). Bonferroni correction was used for *post-hoc* comparison and the magnitude of the Effect size follow the Cohen's rule of thumb: 0.1–0.3: small effect; 0.3–0.5: intermediate effect; 0.5 and higher: strong effect. The ANOVA is used with the assumption that there is a single random error of measurement that is the same for every subject for every trial.

In addition, the Pearson product-moment correlation coefficient, the ICC and the Bland-Altman plot were performed also on the differences between test and re-test condition, while, a Spearman's rank correlation was used for RPE. With this approach, we were able to verify whether the two tests (multi change of direction and RSA) had the same differences, and to compare their sensibility. Finally, a Pearson product-moment correlation coefficient was computed to assess which of the two tests variables (multi change of direction and RSA) had the better relationship with the CMJ, the SJ and the distance performed during the Yo-Yo IR1.

All statistical analyses were performed with the commercial software IBM SPSS version 20.0 (SPSS Inc. Chicago, IL). The level of statistical significance was set at a *P* < 0.05.

## Results

**Figure 2:**
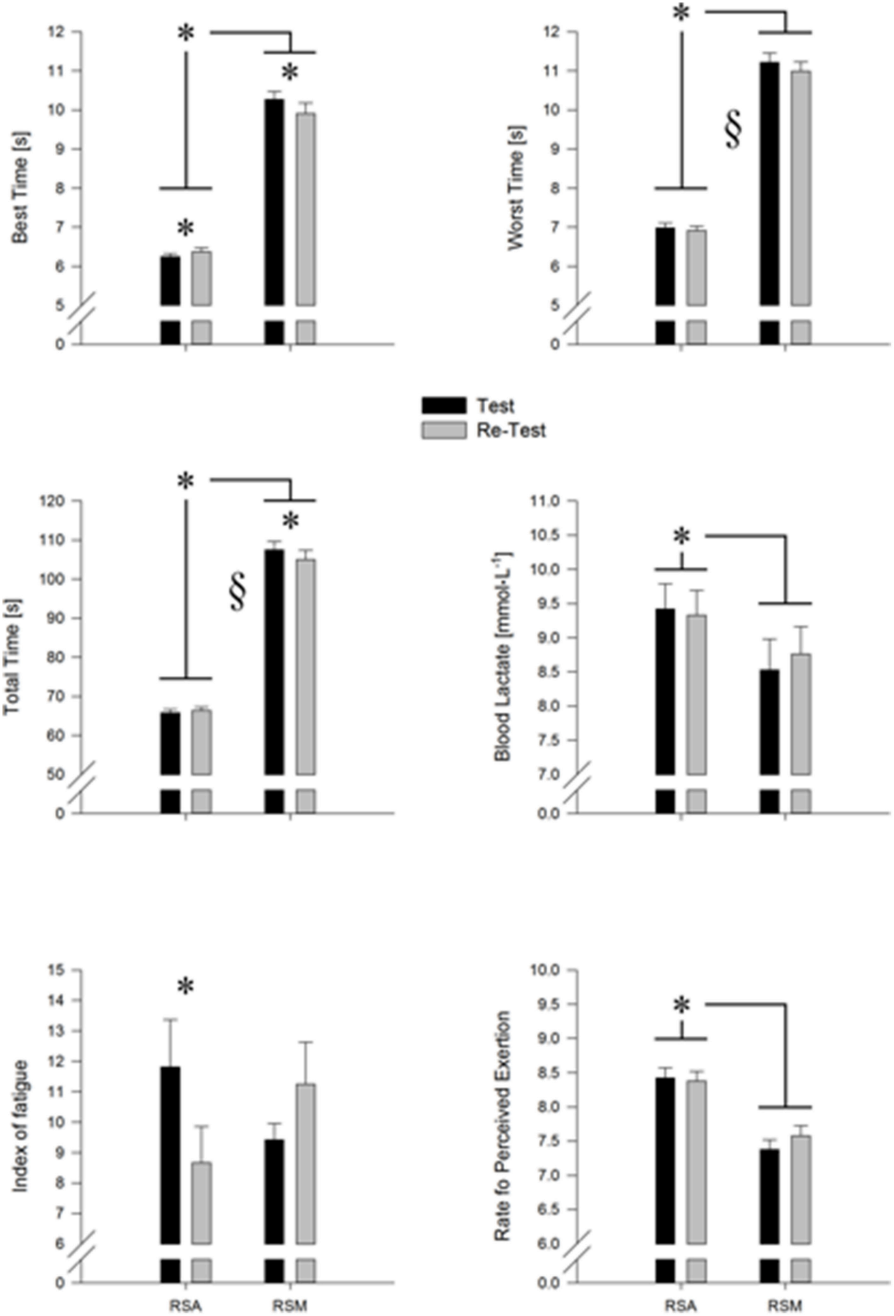
**Graphical representation of the variables studied for repeated sprint ability (RSA) and multi change of direction (RSM) tests**. From the top to bottom are reported: Best time, Worst time, Total time, Blood lactate concentration, Fatigue Index, and the rating of perceived exertion (RPE). The significant value is fixed to *P* < 0.05 and it is reported with an “*” while § indicate the significant difference between test and re-test.

The test and re-test variables of the RSM and the RSA are shown in Figure 2 and, broken down for gender and whole group, are reported in Table 1.

**Table 1:** **Descriptive statistics**.

**Variables**			**RSM**				**RSA**			
			**Mean**	**S.E**.	**95% CI**		**Mean**	**S.E**.	**95% CI**	
BT [s]	Test	Male	10.79	0.34	10.05	11.53	6.57	0.13	6.30	6.84
		Female	9.74	0.25	9.19	10.29	5.93	0.09	5.73	6.14
		Total	10.27	0.21	9.81	10.73	6.25	0.08	6.08	6.42
	Re-Test	Male	10.10	0.43	9.16	11.05	6.75	0.16	6.41	7.08
		Female	9.72	0.32	9.02	10.42	6.01	0.12	5.76	6.26
		Total	9.91	0.27	9.33	10.50	6.38	0.10	6.17	6.59
WT [s]	Test	Male	11.62	0.38	10.79	12.45	7.23	0.21	6.78	7.68
		Female	10.83	0.28	10.21	11.44	6.74	0.15	6.40	7.07
		Total	11.22	0.24	10.71	11.74	6.98	0.13	6.70	7.27
	Re-Test	Male	11.18	0.38	10.35	12.00	7.23	0.17	6.86	7.60
		Female	10.81	0.28	10.20	11.43	6.61	0.13	6.34	6.89
		Total	11.00	0.24	10.48	11.51	6.92	0.11	6.69	7.15
TT [s]	Test	Male	112.25	3.51	104.61	119.90	69.09	1.37	66.11	72.07
		Female	102.62	2.62	96.92	108.32	62.56	1.02	60.34	64.78
		Total	107.44	2.19	102.67	112.21	65.82	0.85	63.96	67.68
	Re-Test	Male	107.50	3.68	99.49	115.51	69.73	1.42	66.64	72.81
		Female	102.60	2.74	96.63	108.57	62.99	1.06	60.69	65.29
		Total	105.05	2.29	100.05	110.04	66.36	0.88	64.44	68.28
Lactate [mmoL]	Test	Male	10.84	0.41	9.95	11.74	8.00	0.61	6.65	9.348
		Female	11.06	0.40	10.17	11.94	7.60	0.61	6.267	8.93
		Total	8.53	0.45	7.53	9.52	9.42	0.37	8.61	10.23
	Re-Test	Male	9.46	0.50	8.35	10.56	7.60	0.75	5.95	9.25
		Female	9.57	0.45	8.58	10.55	7.95	0.67	6.47	9.43
		Total	8.76	0.40	7.87	9.65	9.33	0.36	8.53	10.13
FI	Test	Male	−7.71	0.87	−9.60	−5.82	−10.07	2.48	−15.48	−4.66
		Female	−11.13	0.65	−12.54	−9.72	−13.56	1.85	−17.59	−9.53
		Total	−9.42	0.54	−10.60	−8.24	−11.81	1.55	−15.19	−8.44
	Re-Test	Male	−11.18	2.20	−15.98	−6.39	−7.24	1.92	−11.42	−3.06
		Female	−11.31	1.64	−14.89	−7.74	−10.08	1.43	−13.20	−6.97
		Total	−11.25	1.37	−14.24	−8.26	−8.66	1.20	−11.27	−6.06
RPE	Test	Male	8.20	0.22	7.71	8.69	8.40	0.24	7.88	8.92
		Female	6.56	0.17	6.19	6.92	8.44	0.18	8.06	8.83
		Total	7.38	0.14	7.07	7.68	8.42	0.15	8.10	8.75
	Re-Test	Male	8.60	0.24	8.08	9.12	8.20	0.22	7.71	8.69
		Female	6.56	0.18	6.17	6.94	8.56	0.17	8.19	8.92
		Total	7.58	0.15	7.25	7.90	8.38	0.14	8.07	8.68

Test and re-test variables of the multi-direction repeated sprint ability test (RSM) and the repeated sprint ability test (RSA) divided for gender and whole group (Total), expressed as mean and standard Error (S.E.). Moreover, the number of participants and the 95% of confident interval (CI) of the following variables are reported: BT, Best Time; WT, Worst Time; TT, Total Time; BLa, Blood lactate concentration; IF, Index of fatigue; and RPE, rating of perceived exertion.

The test and re-test variables of the RSM and the RSA broken down for gender and whole group are reported in Table 1.

**Figure 3:**
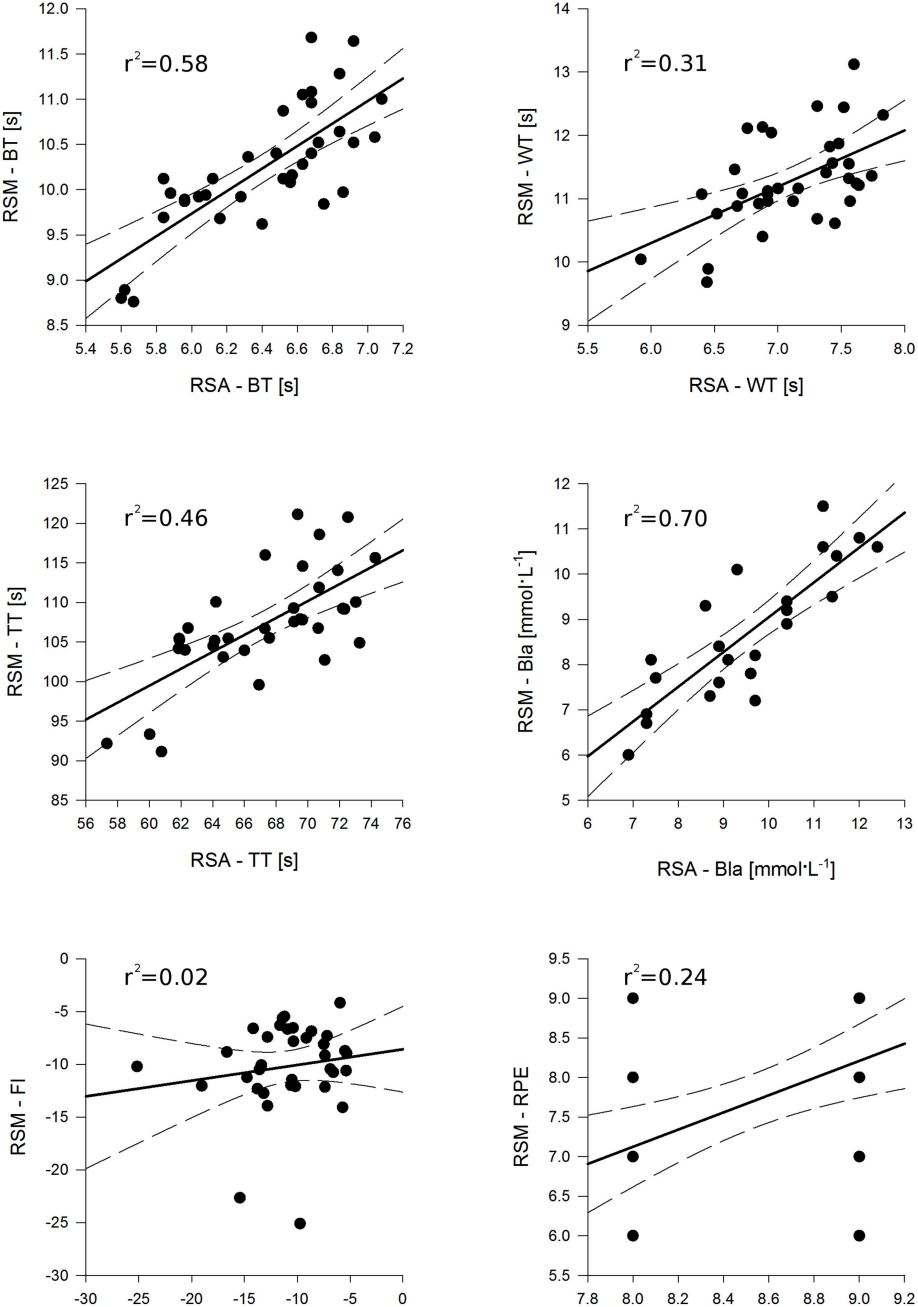
**Correlation between repeated sprint ability (RSA) and multi change of direction (RSM) tests for each variables**. BT, Best time; WT, Worst time; TT, Total time; BLa, Blood lactate concentration; FI, Fatigue Index; and RPE, the rating of perceived exertion. For each graph is reported the regression line (continuous line) and the 95% confident interval (dash line).

The ICCs for the RSA test and RSM test are shown in Table 2 and they were, for almost all the variables, higher than 0.8, showing an agreement from good to excellent. The only variable with acceptable agreement was the BLa of both tests (*r* between 0.6 and 0.8). SEM was lower than 0.257-s for the time variables of the RSA test and of 0.899 mmol·L^−1^ for the BLa, while for the RSM test the SEM values were lower than 0.858-s and of 0.240 mmol·L^−1^, respectively (Table 2). The Bland-Altman results are shown in (Table 2). The biases were all inside the 95% limits of agreement and the bias % were, among tests and variables, lower the 1.5% except for the BLa of both tests that were higher than 10% for the RSM test and higher than 17% for the RSA test respectively. The *z*-value reveled that from the RSM test the BT, BLa and RPE were inside the cut-off of 1.96 (*Z* < |1.96|) meaning that the test and re-test variable were not different, while the other variables WT, TT, and IF were different. The BT, TT, BLa, IF, and RPE of the RSA test were not significantly different between test and re-test. Except the IF, all the variable of the two test were significantly correlated with *r*-value ranging from 0.2 to 0.8 (Table 3, Figure 3). While, the re-test/test differences were all not correlated as well as the ICC were really spread. While a part the BLa all the other variable had the same sensibility as showed by *Z* < |1.96| (Table 3). The results of the ANOVA analyses are reported in the Table 4. All the variables considered, except FI, were statistically different between tests and with a large effect size for the time variables. Overall, WT and TT were lower in the second repetition with an intermediate effect. The gender factor influenced the BT, TT, BLa, and RPE with a small to intermediate effect size. In the first three variables (BT, TT, and BLa), the males were faster and with higher BLa concentration than females while the females reported and higher RPE compare to the males. A significant interaction was found between test and test repetition for the BT, TT, and IF. *Post-hoc* comparison of the BT revealed that the second repetition in the RSA test was slower than the first test (*P* = 0.006), while the second repetition in the RST test was faster than the first test (*P* = 0.024). In the TT *post-hoc* test, the re-test of the RSM test was faster than the test (*P* = 0.001) while, in the IF the re-test of the RSA test had an higher score than the test (*P* = 0.049). The gender factor had an interaction with the two tests (Test × Gender, Table 4) in the BLa and RPE variables. For males subjects, lactate was higher in the RSA test compare to the RSM test (*P* = 0.001) and the female produced more lactate than male in the RSA test (*P* = 0.001). In the RSM test, the RPE was higher for the females compare to males (*P* < 0.0001) while the male perceived more fatigue in the RSA compare to RSM (*P* < 0.001). The TT time was the only significant variable in the interaction test/re-test × Gender: the females were faster in the re-test compare to the test (*P* = 0.001). Moreover, the males were faster in the first test compare to female (*P* = 0.017). Finally, in the triple interaction tests × test/re-test × gender, only the BT, WT, and TT were significant.

**Table 2:** **Test/re-test reliability and agreement between measures**.

**Variables**	**RSM**							**RSA**						
	**ICC**	**SEM**	**Bland altman**					**ICC**	**SEM**	**Bland altman**				
			**Bias**	**Bias %**	**LoA−**	**LoA+**	***Z***			**Bias**	**Bias %**	**LoA−**	**LoA+**	***Z***
BT	0.87	0.12	−0.12	−1.24	−1.17	0.93	−0.94	0.97	0.03	0.08	1.28	−0.25	0.41	0.42
WT	0.93	0.09	−0.03	−0.23	−0.86	0.80	−3.53	0.92	0.05	−0.02	−0.25	−0.52	0.49	−2.93
TT	0.92	0.86	−0.22	−0.21	−7.93	7.49	−3.94	0.98	0.26	0.57	0.85	−1.89	3.04	0.45
Latt	0.77	0.24	−1.23	10.53	−10.17	7.72	−0.81	0.70	0.90	1.69	−17.91	−6.94	10.32	0.53
IF	0.91	1.00	0.12	1.36	−1.64	1.89	1.98	0.92	0.20	−0.13	−1.35	−1.98	1.72	−1.58
RPE	0.92	0.14	0.10	1.31	−1.13	1.32	1.43	0.30	0.13	−0.08	−0.98	−1.37	1.20	−1.60

Test/re-test reliability and agreement between measures for each variable of the multi-direction repeated sprint ability test (RSM) and the repeated sprint ability test (RSA) for BT, Best Time; WT, Worst Time; TT, Total Time; BLa, Blood lactate concentration; IF, Index of fatigue; and RPE, rating of perceived exertion. The reliability is expressed both with the Intra-class Correlation Coefficient (ICC) and the Standard Error of the Mean (SEM), while from the Bland Altman analysis the reliability is derived from the agreement between measures, reporting: the mean bias (Bias) and the percentage bias (Bias %), the upper (LoA+) and lower (LoA−) limits of agreement and the z-values (Z). The two measures are statistically equivalent when the z-value is inside ±1.96, otherwise they are significantly different.

**Table 3:** **Summary of the applied statistics in this investigation**.

**Variables**	**Pearson correlation**		**Pearson correlation**		**Bland altman**				
	**RSA–RSM**	**Sig**.	**Diff RSA–Diff RSM**	**Sig**.	**ICC**	**Bias**	**LoA+**	**LoA−**	**Z**
BT	0.58^**^	<0.0001	0.00	0.955	0.02	−0.37	0.79	−1.53	−0.43
WT	0.30^**^	0.001	0.08	0.334	−0.78	−0.08	0.88	−1.05	−1.56
TT	0.46^**^	<0.0001	0.01	0.797	−0.10	−2.22	3.98	−8.42	−0.38
Latt	0.70^**^	<0.0001	0.04	0.517	−0.49	0.16	2.84	−2.52	2.34
IF	0.03	0.308	0.03	0.545	0.30	−4.60	8.58	−17.79	−0.39
RPE	0.49^**^	0.002	0.41	0.146	0.58	0.14	1.44	−1.16	1.24

Summary of the applied statistics in this experiment: for BT, Best Time; WT, Worst Time; TT, Total Time; BLa, Blood lactate concentration; IF, Index of fatigue; and RPE, rating of perceived exertion. Pearson correlation coefficient between tests and its relative P-value correlation while, for RPE a Spearman correlation coefficient were computed. Sensibility is reported in the Pearson correlation coefficient and Spearman correlation coefficient for RPE of the differences between test and re-test conditions, with its relative P-value and the Intra-class correlation coefficient (ICC). From the Bland Altman analysis the mean bias (Bias) and the percentage bias (Bias %), the upper (LoA+) and lower (LoA−) limits of agreement and the z-values (Z) are reported. The two measures are statistically equal when the z-value is inside ±1.96, otherwise they are significantly different. The significant value is fixed to P < 0.05 and is reported with an “^**^”.

**Table 4:** **Summary of the ANOVA**.

**Variables**	**Tests (RSA vs. RST)**			**Tests** × **Gender**			**Test/Re-test**			**Test/Re-test** × **Gender**			Tests * Test/Re-test			**Test** × **Test/Re-test** × **Gender**			**Gender**		
	***F*_(1, 11)_**	**Sig**.	**η^2^**	***F*_(1, 11)_**	**Sig**.	**η^2^**	***F*_(1, 11)_**	**Sig**.	**η^2^**	***F*_(1, 11)_**	**Sig**.	**η^2^**	***F*_(1, 11)_**	**Sig**.	**η^2^**	***F*_(1, 11)_**	**Sig**.	**η^2^**	***F*_(1, 11)_**	**Sig**.	**η^2^**
BT	616.00	0.000	0.98	0.48	0.502	0.04	0.25	0.627	0.02	1.33	0.27	0.11	42.18	0.000	0.79	20.06	0.001	0.65	5.89	0.034	0.35
WT	526.64	0.000	0.98	0.01	0.922	0.00	6.51	0.027	0.37	1.59	0.23	0.13	0.756	0.403	0.06	3.17	0.102	0.22	3.33	0.095	0.23
TT	542.00	0.000	0.98	0.07	0.796	0.01	6.78	0.025	0.38	10.49	0.01	0.49	19.77	0.001	0.64	13.29	0.004	0.55	5.27	0.042	0.32
BLa	4.59	0.055	0.29	6.69	0.025	0.38	0.48	0.505	0.04	0.00	0.97	0.00	0.08	0.781	0.01	1.24	0.289	0.10	14.75	0.003	0.57
IF	0.70	0.422	0.06	0.16	0.700	0.01	4.04	0.070	0.27	0.03	0.88	0.00	3.83	0.076	0.26	0.00	0.963	0.00	4.24	0.064	0.28
RPE	35.74	0.000	0.77	35.74	0.000	0.77	0.03	0.867	0.00	0.03	0.87	0.00	1.19	0.300	0.10	2.93	0.115	0.21	17.59	0.002	0.62

Summary of the three-way Analysis of Variance (ANOVA): within factors were the two types of tests (RSA and RST) and test/re-test condition, while the between factor was the gender (male and female). For each factor are reported the F-value with the degrees of freedom, the P-value (Sig.) and the partial eta squared (η^2^). The significant value is fixed at P < 0.05.

**Figure 4:**
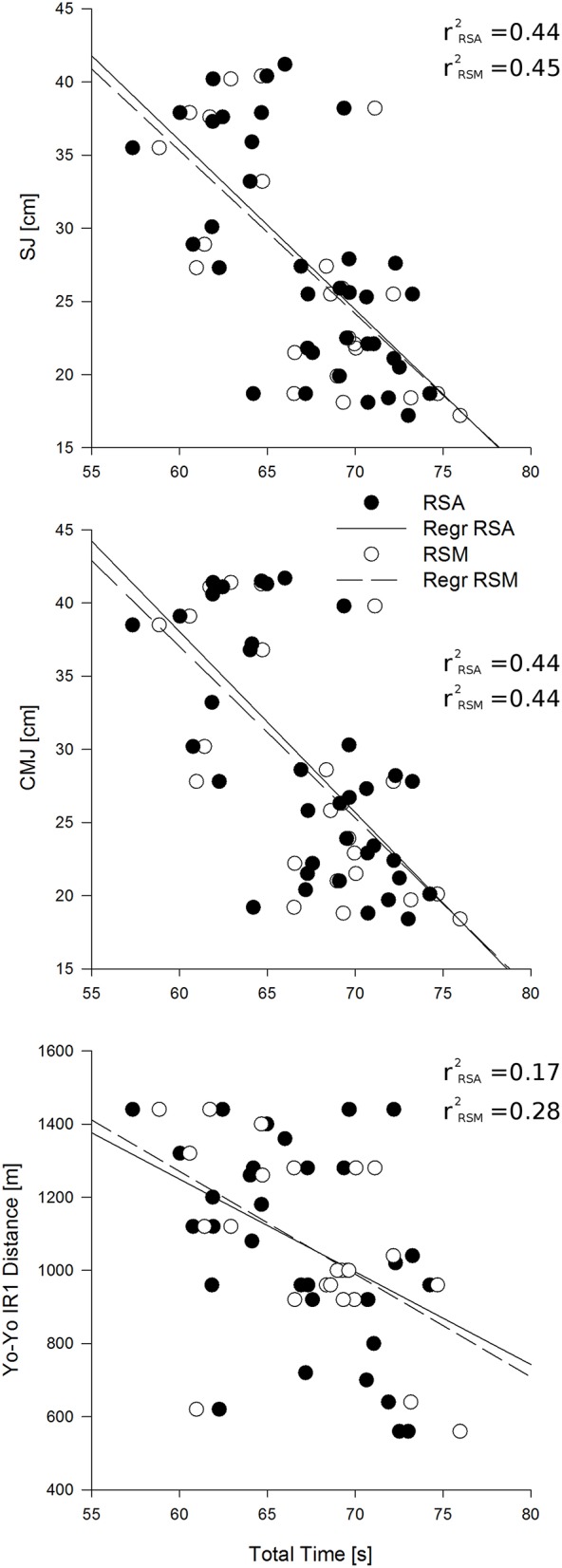
**The first two graphs show the correlation between Total time of the repeated sprint ability (RSA) test (black dots) and the multichange of direction (RSM) test (white dots) with the height of the jump of the squat jump (SJ) (first) and the counter movement jump (CMJ) (second)**. The dashed line is the correlation with the multi change of direction test and the continuous line is the correlation with the repeated sprint ability (RSA) test. The graph above shows the correlation between the Total time of the repeated sprint ability and the multi change of direction tests with the total distance performed during the Yo-Yo Intermittent Recovery Test. The dashed line is the correlation with the multi change of direction (RSM) test while the continuous line is the correlation with the repeated sprint ability test.

Mean SJ was 27.9 ± 7.9 cm, while mean CMJ was 29.42 ± 8.37 cm. All the variables of RSA test were significantly correlated (*P* < 0.05) with both CMJ and SJ (Table 5, Figure 4). All the variables of multi change of direction test, except BLa, were significantly correlated with CMJ and SJ (Table 5, Figure 4). The results of the Yo-Yo IR1 were 1081.11 ± 278.81-m. The BT, WT, and TT of the RSA test were negatively correlated with Yo-Yo IR1 distance, while, BT, WT, TT, and RPE of the multi change of direction test negatively correlated (*P* < 0.05) with Yo-Yo IR1 distance (Table 5). All the other variables did not correlate with CMJ, SJ, nor Yo-Yo IR1 distance.

**Table 5:** **Correlation of the tests with squat jump, counter movement jump and Yo-Yo Intermittent Recovery Test**.

	**RSM–SJ**				**RSA–SJ**			
	***A***	***B***	***r***	**Sig**.	***A***	***b***	***r***	**Sig**.
BT	−7.60	105.82	−0.66	<0.0001^*^	−16.70	135.00	−0.89	<0.0001^*^
WT	−4.48	78.50	−0.41	0.013^*^	−12.38	115.88	−0.71	<0.0001^*^
TT	−0.69	102.10	−0.60	<0.0001^*^	−1.56	133.16	−0.86	<0.0001^*^
BLa	2.04	10.65	0.38	0.0724	2.93	0.66	0.59	0.003^*^
IF	−0.87	19.13	−0.46	0.005^*^	−0.66	20.80	−0.35	0.036^*^
RPE	−5.68	71.78	−0.80	<0.0001^*^	−5.85	77.79	−0.37	0.025^*^
	**RSM–CMJ**				**RSA–CMJ**			
	***A***	***B***	***R***	**Sig**.	***A***	***b***	***r***	**Sig**.
BT	−7.85	109.92	−0.64	<0.0001^*^	−17.45	141.31	−0.88	<0.0001^*^
WT	−4.42	79.34	−0.39	0.022^*^	−12.79	120.22	−0.69	<0.0001^*^
TT	−0.71	105.79	−0.58	<0.0001^*^	−1.62	138.51	−0.85	<0.0001^*^
BLa	2.35	9.32	0.42	0.0480^*^	3.26	−1.02	0.61	0.002^*^
IF	−0.96	19.70	−0.48	0.003^*^	−0.71	21.67	−0.36	0.031^*^
RPE	−6.07	76.31	−0.81	<0.0001^*^	−6.49	84.75	−0.39	0.018^*^
	**RSM–Yo-Yo**				**RSA–Yo-Yo**			
BT	−236.04	3511.57	−0.61	<0.0001^*^	−439.62	3897.24	−0.66	<0.0001^*^
WT	−161.63	2914.24	−0.44	0.007^*^	−327.52	3401.25	−0.54	0.001^*^
TT	−21.86	3436.28	−0.56	<0.0001^*^	−40.23	3783.36	−0.63	<0.0001^*^
BLa	−8.95	1183.07	−0.05	0.802	14.03	990.23	0.09	0.675
IF	−20.54	883.15	−0.33	0.053	−15.48	912.15	−0.24	0.153
RPE	−106.76	1914.99	−0.44	0.007^*^	−17.19	1224.21	−0.03	0.853

The summary of the relation between the variables of the multi-direction repeated sprint ability test (RSM) and repeated sprint ability test (RSA) with the squat jump (SJ), counter movement jump (CMJ) and the total distance of the Yo-Yo Intermittent Recovery Test. For each correlation the intercept (a), the slope (b) and the Pearson correlation coefficient (r) and its relative P-value are reported. The significant value is fixed to P < 0.05 and it is reported with an

* for BT, Best Time; WT, Worst Time; TT, Total Time; BLa, Blood lactate concentration; IF, Index of fatigue; and RPE, rating of perceived exertion.

## Discussion

The main findings of the present study were that:

- multi change of direction repeated sprints was a reliable test, whose reliability was comparable with the that of RSA;
- no difference was found in multi change of direction variables (BT, BLa, IF, and RPE) between test and re-test,
- moderate correlations between multi change of direction and RSA with regards to BT, TT, WT, BLa, and RPE were observed, and
- large correlations of multi change of direction and RSA time variables with performance indices (SJ, CMJ, and Yo-Yo IR1).

The evaluation of multi change of direction test reliability showed that BT, BLa, IF, and RPE yielded same results on two occasions separated by 1 week. Whereas, reliable scores for BT and BLa were expected, the high reliability of FI in multi change of direction was surprising (Tables 1, 2). The reliability of FI has been questioned in the past due to its particular mathematical expression as a drop-off in performance (Oliver, 2009) and other research has shown lower ICC for FI than BT and TT (Austin et al., 2013), which was confirmed by the time variables of RSA in the present study (Table 2). Based on the overall high reliability of multi change of direction, the further use of this novel test was recommended.

Moreover, this recommendation was strengthened by findings on the reliability of multi change of direction's physiological impact (i.e., BLa). Physiological responses (i.e., BLa) following the multi change of direction test resulted in having a different impact when compared with those of the RSA (Table 1). This is not surprising, since it confirms the known evidence that straight sprints with one change of direction and sprint with multiple changes of direction involve mostly separate motor qualities (Padulo et al., 2015b). According to some authors (Buchheit et al., 2010), in terms of shared variance (i.e., coefficient of determination or *R*^2^) between variables, straight sprinting speed and change of direction seem to be separate motor qualities when *R*^2^ < 50%. One main reason for the higher physiological demands of the RSM over the RSA could be that during multi change of direction test players presumably perform higher amounts of high-to-maximal intensity actions than during RSA. In fact, due to the specific nature/design of the multi change of direction, higher numbers of specific power-related actions, such as accelerations, decelerations, changes of directions, occur much more often during the multi change of direction when compared to the RSA. These actions involve intense muscular activity and high muscular efforts (Padulo et al., 2013), which may represent an efficient mean to increase physiological markers of fatigue, as supported by the BLa and RPE values in our study.

Performance in multi change of direction and RSA was similarly correlated with performance in Yo-Yo IR1. The higher correlations observed between RSA and Yo-Yo IR1 should be attributed to the fact that both were performed in one direction, in contrast to multi change of direction which was performed with multi-direction (i.e., five change of directions). A previous study also showed that the scores (mean time and TT) measured in a RSA test presented higher correlation with peak velocity (aerobic power index) determined in an intermittent model including one directional change when compared to a straight line aerobic test (da Silva et al., 2011). Indeed, it is possible to state that the relationship between aerobic fitness and RSA is dependent on the protocol type, thus providing important implications in the design of training models aimed at combining exercise training with the CSR. Although several differences were revealed with regards to the magnitude of these correlations compared to the findings of previous studies, all were in agreement that the stronger correlation was for BLa, followed by BT, TT, WT, and RPE.

Similar correlations were found between both repeated sprint tests (i.e., RMS and RSA) and jump performances (i.e., SJ and CMJ), suggesting that leg muscle qualities such as power is an important determinant of a single or multi change of direction ability (Attene et al., 2014; Padulo et al., 2015c). Despite the importance of muscle power on both types of repeated sprints, it is important to highlight that on multi change of direction (RSM) other aspects as movement technique, agility, and reactive strength are related to performance (Brughelli et al., 2008). Consequently, vertical jump performance seems to be more correlated with linear sprints and drills with single change of direction where higher acceleration are achieved (Attene et al., 2014, 2015b). Higher acceleration produces maximal speeds in less amount of time thus leading to better sprint performances over short distances. Additionally, from a physiological perspective, the obtained relationship between RSM, RSA and vertical jump performances are likely attributable to the similar contribution of the energy systems involved when performing these tasks (Attene et al., 2014). In fact, short sprinting and jumping capabilities are classified as power-type athletic activities, both recognizing the phosphocreatine metabolism as the mean energy supplier (Chamari and Padulo, 2015). Furthermore, depletion of phosphocreatine stores is usually considered as a limiting factor for the RSA and vertical jump performances.

A limitation of the present study concerns the generalizability of its findings in populations with other performance characteristics. Coaches and fitness trainers, who wish to apply these findings, should take into account the relatively heterogeneous (including both males and females) sample of basketball players that participated, which might impact on the magnitude of correlations. In the case of elite basketball players which consists of a relatively homogeneous group (e.g., shorter range of jumping performance or aerobic capacity), these correlations might be weakened. Therefore, the novel multi change of direction test should be examined for validity and reliability in other samples of basketball players differing for age, sex, and competition level. Further, it may be worth doing longitudinal studies wherein same subjects should be tested over months or years. Correlation analysis for longitudinal changes in each variable could be helpful for eliminating any between-subject difference and give more strength to our present results.

In conclusion, based on the findings of the present study, it was concluded that the novel test RSM was valid and reliable. This measure of sport-specific RSA was recommended for further use in young basketball players, where coaches and fitness trainers might apply it to monitor performance.

## Author contributions

JP conceived the experiment, JP, PN, AD, GA, JD, AZ, MO, GM collected the data. NB and FB analyzed the data. All authors wrote and approved the manuscript.

### Conflict of interest statement

The authors declare that the research was conducted in the absence of any commercial or financial relationships that could be construed as a potential conflict of interest.
